# Cross-diagnostic validity of the Nottingham health profile index of distress (NHPD)

**DOI:** 10.1186/1477-7525-6-47

**Published:** 2008-07-02

**Authors:** Christine Wann-Hansson, Rosemarie Klevsgård, Peter Hagell

**Affiliations:** 1 Faculty of Health and Society, Malmö University, SE-205 06, Malmö, Sweden; 2 Department of Health Sciences, Lund University, PO Box 157, SE-221 00, Lund, Sweden; 3 Department of Neurology, Lund University Hospital, SE-221 85, Lund, Sweden

## Abstract

**Background:**

The Nottingham Health Profile index of Distress (NHPD) has been proposed as a generic undimensional 24-item measure of illness-related distress that is embedded in the Nottingham Health Profile (NHP). Data indicate that the NHPD may have psychometric advantages to the 6-dimensional NHP profile scores. Detailed psychometric evaluations are, however, lacking. Furthermore, to support the validity of the generic property of outcome measures evidence that scores can be interpreted in the same manner in different diagnostic groups are needed. It is currently unknown if NHPD scores have the same meaning across patient populations. This study evaluated the measurement properties and cross-diagnostic validity of the NHPD as a survey instrument among people with Parkinson's disease (PD) and peripheral arterial disease (PAD).

**Methods:**

Data from 215 (PD) and 258 (PAD) people were Rasch analyzed regarding model fit, reliability, differential item functioning (DIF), unidimensionality and targeting. In cases of cross-diagnostic DIF this was adjusted for and the impact of DIF on the total score and person measures was assessed.

**Results:**

The NHPD was found to have good overall and individual item fit in both disorders as well as in the pooled sample, but seven items displayed signs of cross-diagnostic DIF. Following adjustment for DIF some aspects of model fit were slightly compromised, whereas others improved somewhat. DIF did not impact total NHPD scores or resulting person measures, but the unadjusted scale displayed minor signs of multidimensionality. Reliability was > 0.8 in all within- and cross-diagnostic analyses. Items tended to represent more distress (mean, 0 logits) than that experienced by the sample (mean, -1.6 logits).

**Conclusion:**

This study supports the within- and cross-diagnostic validity of the NHPD as a survey tool among people with PD and PAD. We encourage others to reassess available NHP data within the NHPD framework to further evaluate the strengths and weaknesses of this simple patient-reported index of illness-related distress.

## Background

The Nottingham Health Profile (NHP) is a widely used 6-dimensional (energy, pain, emotional reactions, sleep, social isolation, and physical mobility) generic health status questionnaire [1]. The NHP has undergone extensive evaluation and both strengths and weaknesses have been demonstrated [2]. A commonly observed limitation of the NHP has been skewed score distributions with large ceiling and, particularly, floor effects [3-5]. This complicates interpretation of extreme scores and impairs the ability to detect changes and differences. Furthermore, some of the NHP domains have relatively few (3 to 5) dichotomous items. This limits the precision of scores [6-8].

The NHP index of Distress (NHPD) is a 24-item measure of illness-related distress embedded in the NHP [9]. While it has not been extensively used or evaluated, available data have shown promise and suggest that it can provide a unidimensional measure of illness-related distress [4,10-12]. Indeed, the NHPD has the potential, at least in part, to overcome limitations associated with NHP domain scores. The larger number of items should improve reliability and precision of scores. Accordingly, available studies have shown less floor/ceiling effects and indicated better responsiveness and reliability of the NHPD than the six NHP domain scores [4,9-12]. However, its generic properties, i.e. whether scores can be interpreted the same way across different diagnoses, remain to be determined. This is particularly important because a main assumption and theoretical advantage with generic outcome measures is the possibility to make valid comparisons across patient groups. Support for these properties is gained when scales work the same way and have the same meaning in different groups. This can be assessed by analyzing the presence of differential item functioning (DIF) [13,14].

Generic outcome measures can be more or less suitable for certain groups of people. As such, the NHP has been found to work best with chronic, disabling conditions, and with elderly populations who are likely to have at least some of the problems represented in each of its six domains [2]. Parkinson's disease (PD) and peripheral arterial disease (PAD) exemplify two chronic disabling disorders associated with aging where the NHP has been commonly used [4,11,15-18]. PD is a chronic progressive neurodegenerative condition characterized by motor symptoms such as bradykinesia, rigidity and resting tremor. However, non-motor features such as fatigue, depression, sleep disturbances, pain and autonomic dysfunctions are also frequent and a common source of disability [19]. PAD is associated with a wide spread arterial disease and significantly increased risk of stroke, myocardial infarction and cardiovascular death. Symptoms range from leg pain while walking to severe pain in the limb also at rest, non-healing ulcers and gangrene [20]. Besides pain and restricted mobility, fatigue, emotional distress and sleep disturbances are common problems in PAD [18]. PD and PAD therefore appear to represent suitable diagnostic groups for assessing the NHPD and explore its cross-diagnostic validity and comparability.

The Rasch measurement model [21] offers particular advantages over traditional psychometric methods in evaluating measurement scales [8,22,23]. The model rests on a mathematical definition of the requirements for linear measurement, which is achieved when data accord with model specifications. Because the model articulates measurement requirements, sources of violations to model assumptions are sought and adjusted for in the data rather than trying to fit another model [24]. Rasch analysis thus determines the extent to which observed data conform with model specifications and provides a powerful means of assessing a scale's measurement properties, including DIF [14,23,25-28].

This study assessed the measurement properties and cross-diagnostic validity of the NHPD as a survey instrument among people with PD and PAD.

## Methods

### Samples

Data from people with PD were taken from three sources: postal survey data from patients receiving care at a neurology department (n = 71) [4], consecutive patients fulfilling criteria for neurosurgical interventions for PD (n = 26) [29], and consecutive PD outpatients without other significant disorders (n = 118) [30] (Table 1). All PD patients had a neurologist diagnosed PD [31] and two of the original samples [4,30] provided ratings (mild, moderate or severe) of their overall perceived severity of PD [32].

**Table 1:** Sample characteristics

	PAD (*n *= 258)	PD (*n *= 215)	P-value
Age, mean (SD)	69 (10.2)	65 (9.9)	.000^a^
Sex (% male/female)	57/43	57/43	.980^b^
Severity of disease, n (%)			
Intermittent claudicatio	141 (55.0)	NA	
Critical limb ischemia	117 (45.0)	NA	
Perceived PD severity, n (%) ^c^			
Mild	NA	37 (20.0)	
Moderate	NA	118 (63.0)	
Severe	NA	33 (17.0)	
NHPD, md (q1–q4)	20.8 (8.3–37.5)	16.7 (4.2–29.2)	.002^a^

^a ^Mann-Whitney U-test.

^b ^Chi-square test.

^c ^As rated by a subset of 188 patients [4,30].

PAD, peripheral arterial disease; PD, Parkinson's disease; SD, standard deviation; NHPD, Nottingham Health Profile index of Distress; md, median; NA, not applicable.

PAD data were taken from two different sources: data from 168 [16] and 90 [5] consecutive patients admitted for treatment of lower limb ischemia at vascular surgical units and without other diseases compromising their walking capacity (Table 1). The severity of ischemia was documented according to standards for grading lower limb ischemia [33].

All original studies had cross-sectional designs and were approved by the respective local research ethics committees.

### NHP index of Distress (NHPD)

The NHPD was devised from the NHP, specifically omitting items relating to physical disability and items precluding its use in hospitalized patients [9]. It consists of 24 dichotomous ("yes"/"no") items that yield a score ranging between 0 and 24, with higher scores indicating more distress. In this study, the NHPD was derived from the full 38-item NHP (Swedish version [34]), as self completed either at home [4], during study visits at the clinic [29,30] or at admission to hospital [5,16].

### Rasch analysis

The Rasch model [21,22] is a probabilistic measurement model that separately locates persons and items on a common linear logit (log-odd units) metric, which ranges from minus infinity to plus infinity (with mean item location set at zero). Locations along the logit scale reflect how much of the measured construct that is represented and possessed by each item and person, respectively, as estimated from response patterns. When data accord with the model, Rasch derived measures have the same meaning throughout the range of measurement and the relative locations of any two items (or persons) are independent of the locations of other items or persons. Furthermore, different subsets of the same class of items (or persons) give equivalent location estimates. These features distinguishes the Rasch model from other approaches such as classical test theory, 2- and 3-parameter item response theory models [8,23,24].

The Rasch model assumes that the scale is unidimensional, i.e., that items tap a common underlying latent trait, and that items are locally independent, i.e., the response to one item should be independent of responses to other items. These aspects are reflected in the fit of data to the model [22,35], which can be assessed for each item by dividing the sample into class intervals according to their locations on the measured construct. Accordance between class interval responses and model expectations (represented by the item characteristic curve, ICC) is then studied graphically as well as quantitatively, using standardized residuals (should range between -2.5 and +2.5) and their associated chi-square statistics (should be non-significant) [22,35]. In general, large negative residuals signal local dependency and large positive values indicate violation of unidimensionality. In addition, overall fit is reflected in the mean and standard deviation of the residuals (expected values of 0 and 1, respectively) and the total item-trait interaction chi-square statistic (expected P-value > 0.05).

Differential item functioning (DIF) is an additional aspect of model fit and occurs when subgroups of people at *comparable levels *on the measured construct respond systematically differently to items [13]. DIF can produce biased scores, thereby challenging the validity of comparing data across subgroups, and may reflect or threaten unidimensionality [36]. DIF can either be uniform (item responses differ uniformly between groups across class intervals) or non-uniform (group differences vary across class intervals) [14,26]. Uniform DIF can be adjusted for by splitting the item into two new items, one for each subgroup, whereby the bias is controlled for while the information from the item is retained [14].

Unidimensionality can be further assessed based on a principal component analysis (PCA) of residuals and an independent t-test approach that compares estimates of person locations based on different item subsets [37,38]. If deviation from unidimensionality is trivial, the number of person locations that differ between the two item sets is small.

### Analysis plan

The NHPD was Rasch analyzed using the RUMM2020 software (Rumm Laboratory Pty Ltd., Perth). We first examined the fit of the NHPD within each of the two diagnostic groups separately by dividing the samples into three class intervals with 57–61 (PD) and 68–74 (PAD) people in each. Next, the samples were pooled and divided into six class intervals with 51–78 people in each before examination of model fit, reliability, and DIF by diagnosis. If DIF was identified, this was adjusted for by splitting items into disease specific items followed by re-analyses of measurement properties. Due to the large number of statistical tests, P-values were interpreted as significant at the 0.05 level following Bonferroni correction [39].

The clinical significance of any observed DIF was studied by assessing if DIF influenced the estimated person locations (logit measures). First, the person locations obtained after adjustment for DIF were compared to those estimated from the non-DIF-adjusted scale. Before doing so, items without DIF in the original scale were anchored by their item locations from the DIF-adjusted scale to assure that the two sets of person estimates measured on the same metric. The two sets of person locations were then plotted and correlated with each other to assess the influence of DIF on people's estimated distress levels. Second, we tested whether the same total scores reflected the same levels of distress across samples [27]. In this procedure one item block was created for each diagnosis and arranged next to each other with missing values recorded as responses from people with PD to the PAD specific item block, and vice verse. A third, vertical block of items contains the item responses for both diagnoses together, thus providing linkage in the dataset. The three item blocks were then treated as multiple tests and the logit values of the same summed raw scores were compared across the samples [27].

To assess unidimensionality, two sets of person locations were produced; one from the items with the largest (≥ 0.3) positive residual loadings on the first principal component and one from items with the largest negative loadings [38]. This was followed by independent t-tests of the two estimated locations for each person. Unidimensionality was considered statistically supported when the proportion of significant individual t-tests, or the lower bound of the associated 95% binomial confidence interval, did not exceed 0.05 [38].

Finally, we assessed how well the best fitting unidimensional NHPD solution accorded with the levels of illness-related distress experienced by the sample.

## Results

Raw NHPD scores covered the full range (0–24) in the PAD sample (median, 5; q1–q3, 2–9) and ranged between 0 and 21 (median, 4; q1–q3, 1–7) in the PD sample. The median in the combined sample was 5 (q1–q3, 2–8).

### Within-diagnoses analyses

Within-diagnoses Rasch analyses showed good overall model fit in both PD (item residual mean [SD], -0.402 [1.191]; item-trait interaction, P = 0.077) and PAD (item residual mean [SD], -0.512 [1.064]; item-trait interaction, P = 0.164). Reliabilities were 0.848 (PD) and 0.838 (PAD). There was no significant item level misfit in either of the samples.

### Pooled data and cross-diagnoses validity

The NHPD displayed good reliability and overall fit to the measurement model (Table 2). At the item level, item 9 displayed a non-significant (following Bonferroni adjustment) but relatively large negative fit residual value and a somewhat large chi-square value relative to the other items (Table 3). No other items showed signs of misfit (Table 3).

**Table 2:** Overall Rasch model fit statistics and reliability of the NHPD

	Original NHPD	NHPD adjusted for DIF ^d^
*Item fit residual*		
Mean ^a^	-0.571	-0.488
SD ^b^	1.416	1.250
*Total item-trait interaction*		
Chi-square (df)	134.337 (120)	188.538 (155)
P-value	0.175	0.034
Reliability ^c^	0.841	0.844

^a ^Should be close to 0 [35].

^b ^Should be close to 1 [35].

^c ^Index of person separation, a Rasch based reliability statistic analogous to Cronbach's alpha/KR-20 [22,35]. Indicates the degree to which people can be separated into discrete groups. Values of 0.7 and 0.8 are the minimum required to discern two and there groups, respectively [44].

^d ^Items 4, 6, 7, 8, 11, 17 and 18 split by diagnosis.

NHPD, Nottingham Health Profile index of Distress; DIF, differential item functioning; SD, standard deviation; df, degrees of freedom.

**Table 3:** Rasch item and fit statistics for the NHPD^a^

		Item statistics ^c^		Fit statistics		
Item ^b^		Location	SE	Residual ^d^	Chi square ^e^	P-value

1 (1)	Tired all the time	-1.154	0.116	1.383	9.057	0.10681
2 (2)	Pain at night	-0.97	0.113	1.914	8.695	0.121867
3 (3)	Things get me down	-0.732	0.116	-1.841	5.612	0.345829
4 (4)	Unbearable pain	0.425	0.14	-1.025	2.982	0.702838
5 (6)	Joy forgotten	0.231	0.134	-2.03	9.32	0.09695
6 (7)	Feeling on edge	-1.127	0.112	0.257	4.383	0.495644
7 (8)	Painful to change position	-1.874	0.113	1.964	2.8	0.73085
8 (9)	Feel lonely	0.297	0.136	0.521	2.791	0.732098
9 (12)	Everything is an effort	-0.95	0.113	**-3.107**	13.87	0.016456
10 (16)	Days seem to drag	0.617	0.146	-1.448	3.529	0.619006
11 (20)	Losing temper easily	-0.409	0.12	1.321	7.45	0.189241
12 (21)	Feel close to nobody	1.302	0.177	-0.336	6.317	0.276545
13 (22)	Lie awake most of night	1.269	0.174	-1.313	1.735	0.884403
14 (23)	Feel as if losing control	1.05	0.164	-1.557	9.758	0.082392
15 (26)	Soon run out of energy	-1.724	0.111	-1.318	5.277	0.383036
16 (28)	In constant pain	-0.356	0.12	-1.228	5.276	0.38318
17 (29)	Takes long to get to sleep	-0.485	0.118	0.928	4.264	0.512122
18 (30)	Feel like a burden	0.075	0.13	-1.224	3.12	0.681545
19 (31)	Kept awake by worries	0.904	0.157	-1.776	5.506	0.357274
20 (32)	Life not worth living	0.815	0.153	-1.759	5.433	0.365301
21 (33)	Sleep badly at night	-0.966	0.113	0.799	1.207	0.944184
22 (34)	Hard to get on with people	3.458	0.401	-0.896	1.985	0.85118
23 (37)	Depressed when waking up	0.263	0.134	-2.121	6.171	0.28995
24 (38)	In pain when sitting	0.042	0.129	0.193	7.798	0.167709

^a ^Performed with the sample divided into six class intervals according to person locations on the measured variables.

^b ^Original NHP item numbers in parenthesis.

^c ^Expressed in linear log-odds units (logits), with mean item location set at 0.

^d ^Residuals summarize the deviation of observed from expected responses. Deviation from the recommended [35] range of -2.5 to +2.5, indicating item misfit, are bold.

^e ^Higher values represent larger deviations from model expectations.

NHPD, Nottingham Health Profile index of Distress; SE, standard error.

**Figure 1:**
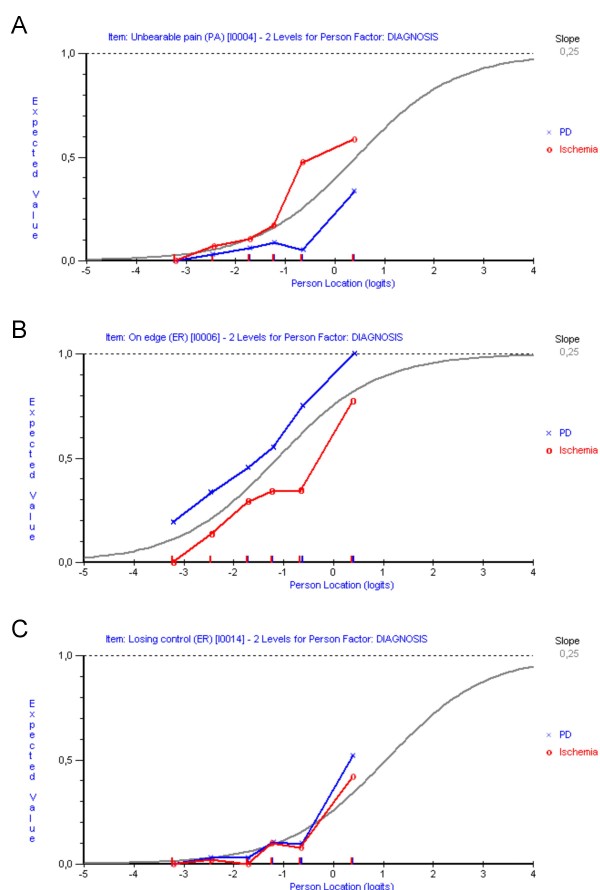
**Differential item functioning (DIF) between people with PD and PAD**. Examples of two NHPD items (panel A, item 4/"unbearable pain"; panel B, item 6/"feeling on edge") displaying cross-diagnostic DIF. The item characteristic curves (ICCs; grey curves) represent the expected probabilities of item endorsement (y-axis) at various levels of the measured construct (x-axis). Superimposed plots represent the observed responses by people with PD and PAD, as divided into six class intervals according to their levels of illness-related distress. Observed differences indicate that items do not work the same way in the two diagnostic groups. For comparison, panel C illustrates an item without DIF (item 14/"feel as if losing control").

DIF analyses identified uniform DIF by diagnosis for seven items (Table 4; Fig. 1). After splitting these items into two each (one for PD and one for PAD) the overall item-trait interaction was somewhat significant (P = 0.03), whereas the overall item residual mean and standard deviation, as well as reliability, showed some improvement (Table 2). This pattern was similar also when considering fit statistics after successive splitting of each item one at a time. That is, fit residual means and standard deviations, as well as reliability, displayed various degrees of improvements whereas chi-square values and their associated p-values did not [see Additional file 1].

**Table 4:** NHPD items with uniform DIF by diagnosis (PD vs PAD) ^a, b^

Item ^c^		F-value ^d^	P-value	DIF direction ^e^
4 (4)	Unbearable pain	15.32361	0.000107	PAD > PD
6 (7)	Feeling on edge	32.24345	0.000000	PD > PAD
7 (8)	Painful to change position	23.15150	0.000004	PAD > PD
8 (9)	Feel lonely	10.95699	0.001024	PD > PAD
11 (20)	Losing temper easily	28.24274	0.000000	PD > PAD
17 (29)	Takes long to get to sleep	26.16763	0.000000	PAD > PD
18 (30)	Feel like a burden	12.83750	0.000385	PD > PAD

^a ^Performed with the sample divided into six class intervals according to person locations on the measured variables.

^b ^Nonuniform DIF was not detected.

^c ^Original NHP item numbers in parenthesis.

^d ^Analyses of variance of deviations from model expectation along the latent trait across people with PD and PAD.

^e ^Direction of observed DIF, PAD > PD indicates higher probability for people with PAD to endorse an item, and vice verse.

NHPD, Nottingham Health Profile index of Distress; DIF, differential item functioning; PD, Parkinson's disease; PAD, peripheral arterial disease.

**Figure 2:**
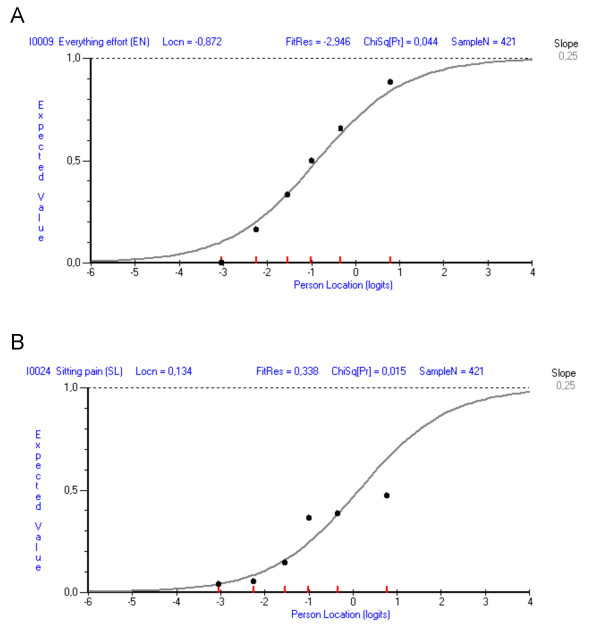
**Two items with some signs of misfit in the DIF-adjusted NHPD**. Item characteristic curves (ICCs) of items 9 ("everything is an effort"; panel A) and 24 ("in pain when sitting"; panel B) following scale adjustment for cross-diagnostic DIF. Black dots represent the observed responses in the sample as divided into six class intervals according to their levels of illness-related distress, indicated by red marks on the x-axis.

After splitting the seven DIF associated items the negative residual for item 9 remained relatively large (-2.946) but non-significant. Inspection of the class interval plots relative to the ICC of item 9 indicated that the overall deviation from expectation primarily concerned the least distressed class interval (Fig. 2A). Other individual item fit residuals were not significant (range, -2.007 to 2.306). However, item 24 showed a relatively large chi-square value (14.019) compared to the other items (range, 1.024–11.396), although its fit residual value was good (0.338) (Fig. 2B).

An attempt was made to improve the measure by omitting items 9 and 24 from the DIF adjusted scale. Both resulted in improved and non-significant overall item-trait interaction statistics (omitting item 9: P = 0.133; omitting item 24: P = 0.194). However, the overall residual means and standard deviations did not improve (omitting item 9: mean [SD], -0.498 [1.171]; omitting item 24: mean [SD], -0.494 [1.282]) and reliability decreased slightly (omitting item 9: 0.831; omitting item 24: 0.840). Similarly, when both items 9 and 24 were deleted the item-trait interaction improved (P = 0.353) whereas the overall residual mean (-0.506), standard deviation (1.228) and reliability (0.828) did not. No additional DIF or individual item misfits were detected in either of these analyses.

Taken together, these analyses showed good model fit but DIF by diagnosis for the original NHPD, modest signs of misfit after adjusting for DIF, and lack of unequivocal improvement of fit following item deletion. Given these observations in combination with clinical considerations, it was decided to assess the clinical significance of observed DIF based on all 24 NHPD items.

**Figure 3:**
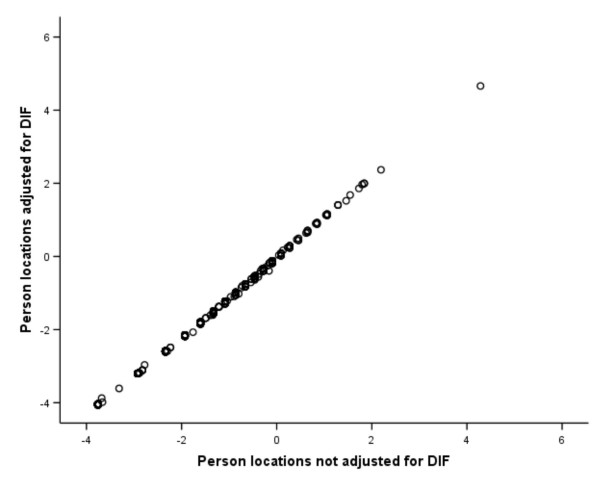
**Impact of DIF on person measures**. Scatterplot of locations (logit measures) of each person estimated from the NHPD after adjustment for DIF by means of item split (y-axis) compared to those obtained from the original items not adjusted for DIF but anchored by DIF-free item calibrations from the DIF-adjusted scale (x-axis).

**Figure 4:**
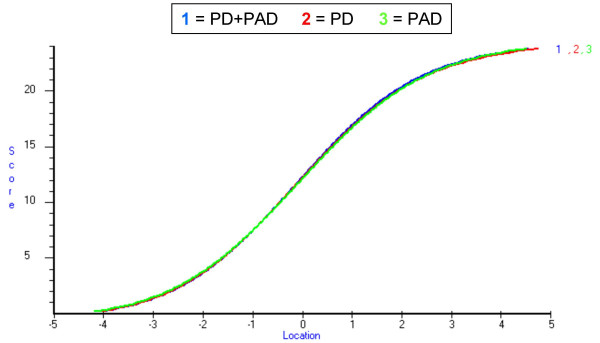
**Total NHPD scores and their corresponding logit measures**. Comparison of raw total NHPD scores' (y-axis) logit values (x-axis) from the combined PD+PAD sample (curve 1, blue) and with each item treated as a diagnostic specific item (curves 2 and 3, red and green).

Plots of estimated person levels of illness-related distress derived from items with and without adjustment for DIF were virtually identical (Fig. 3) with Pearson and intra-class correlations of 1.0 and 0.99, respectively. We then tested whether the same total scores reflected the same levels of distress across samples by examining the equivalence of raw scores-to-locations estimates between diagnosis specific and common item sets. The results showed virtually no differences (Fig. 4).

PCA of residuals showed that the first principal component explained 13% of the total variance among residuals in the original NHPD and 11% of the total variance in the DIF-adjusted scale. Using independent t-tests, person location estimates based on items with large (> 0.3) positive and negative loadings on the first principal component were compared. When only respondents without minimum or maximum scores on the two subsets of items were taken into account the proportions of significant t-tests from the DIF-adjusted and the non-DIF-adjusted NHPD were 0.008 and 0.037, respectively. When the full sample was taken into account the proportions of different estimates for the DIF-adjusted and the non-DIF-adjusted scales were 0.064 and 0.081 (lower 95% CI bounds, 0.04 and 0.06), respectively. This suggests some degree of multidimensionality in the non-DIF-adjusted scale.

**Figure 5:**
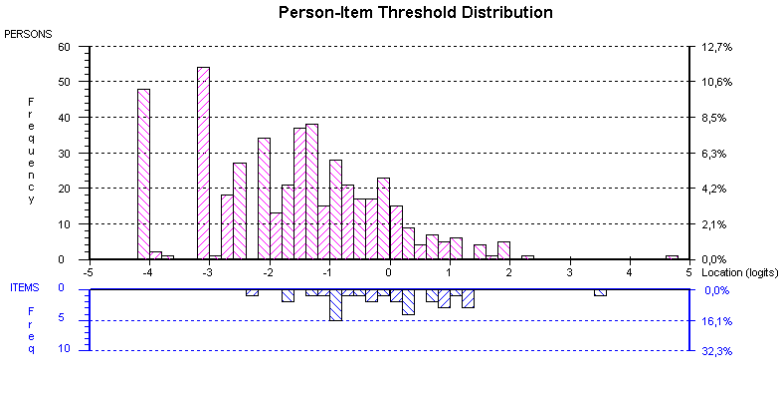
**Targeting**. Distribution of the locations of people (upper panel) and NHPD items (lower panel) on the common logit metric (negative values = less illness-related distress) following adjustment for DIF.

Figure 5 depicts the distribution of persons relative to items for the DIF-adjusted NHPD. The mean (SD) person location was -1.619 (1.454), meaning that the items represent more distress than that experienced by the sample. In terms of raw score floor and ceiling effects of the original NHPD, 48 people who responded to all 24 items scored 0 (10% floor effect), and another 3 people (0.06%) with missing item responses (range, 1–10) scored 0 based on the items they had responded to. One person who responded to all 24 items scored maximum (0.2% ceiling effect).

## Discussion

The aim of this study was to evaluate the measurement properties and cross-diagnostic validity of the NHPD as a survey tool among people with PD and PAD. We found that the NHPD displayed generally good measurement properties but signs of DIF by diagnosis for seven items. However, this DIF did not impact the total score, thus supporting the generic measurement properties of the NHPD among people with PD and PAD.

The most important observation from this study is that observed DIF cancelled out and was not found to have any meaningful effects on the total NHPD score. This conclusion is based on the observation that estimated person locations were virtually identical regardless of whether DIF was adjusted for or not, and the linear measures corresponding to different raw total scores were also very similar. The approach employed here to assess the impact and clinical significance of DIF on the total score is reasonable because the total raw score is directly related to, and a sufficient statistic for estimation of, the linear measure of a person [35]. These results provide empirical support for the assumed generic properties of the NHPD. However, additional studies in other target populations are needed to generalize these conclusions.

Overall model fit did not improve but showed signs of deterioration following adjustment for cross-diagnostic DIF. This may be considered somewhat surprising given that DIF violates model assumptions [22]. However, DIF represents an aspect of model fit additional to that provided by residual based assessments across class intervals. One possible explanation for the significant item-trait interaction statistic following item splits may be that the observed DIF were signs of multidimensionality rather than "true" DIF among these items. This view is supported by the lack of improved overall fit following item split and signs of multidimensionality in the independent t-test protocol (see below). An additional explanation could be that item 24 displayed some signs of misfit in the DIF-adjusted scale, although removing this item did not lead to unequivocal improvements. The statistically significant item-trait interaction statistic also needs to be interpreted in view of the sample size [35,40]. If, for example, the sample studied here had consisted of ten people less, this statistic would not have been significant. Taken together, we therefore consider the statistically significant item-trait interaction not to be of any greater practical significance. Similarly, it also appears reasonable to retain items 9 and 24 since the observed misfit largely stemmed from one (item 9) or two (item 24) class intervals. Furthermore, these items behaved well otherwise and their removal did not result in unequivocal scale improvements.

The independent t-test protocol [37,38] identified signs of multidimensionality in the scale when not adjusted for DIF. However, given that this finding was just marginally significant (lower 95% CI bound, 0.06) it may be argued whether this is of any practical concern. Indeed, this test, as any other statistical test [40], is dependent on sample size. If, for example, half or two thirds of the current sample size had been used instead (with the same proportion of significant individual t-tests), the statistical conclusion would have supported unidimensionality. Therefore, although the independent t-test protocol appears more useful in detecting multidimensionality than residual based fit indices and factor analytic approaches [37,38] it must be borne in mind that this, in itself, also is a somewhat arbitrary test. Inferences are dependent on and, therefore, differ according to sample sizes [40]. Other aspects of this test also need to be considered. First, although often considered non-problematic with sample sizes above 200 [41], methods such as PCA assumes that data are normally distributed. Secondly, the rationale for the suggested loading of 0.3 as a cut-off to define items to be included in the independent t-test protocol [38] is unclear and other criteria could also be conceivable; additional studies regarding the optimal approach to using this test are warranted. Unidimensionality is not an absolute but a relative matter and there is no single agreed-upon method to test unidimensionality. Therefore, the decision whether a scale is sufficiently unidimensional should ultimately come from outside the data and be driven by the purpose of measurement and clinical/theoretical considerations [22].

In accordance with expectations and previous observations [4,10,12] we found the NHPD to display considerably less floor effects than the original NHP dimension scores typically have and that the observed proportion met the suggested 15% criterion [42]. This is an important observation because large floor and ceiling effects impact the possibility to differentiate between respondents and detect changes over time [43]. However, examination of the distribution of persons and items in this study revealed that a proportion of people exhibited levels of distress that were lower than that covered by the NHPD items. The implication of this observation is that those people are measured with less precision and confidence, which impacts the ability of the scale to reliably detect differences and changes in this region of the outcome space. However, the NHPD was still able to distinguish among three different strata of people, as indicated by reliabilities above 0.8 [44], and experiences from clinical trials in PD and post-acute inpatient care [11,45,46] have provided general support for its responsiveness.

Although the NHPD appears more useful than the six-dimensional NHP, our observations are in general agreement with recommendations for the NHP [2] and suggest that the NHPD probably is most suitable for studies of people with chronic, disabling conditions expected to experience relatively high levels of distress. The suitability of a scale relates to the purpose of its use. For example, our observations suggest that the NHPD would not be suitable for a clinical trial targeting people experiencing relatively mild disease impact, whereas there is support for its usefulness in trials aimed at more severely affected individuals. The NHPD also appears useful for survey purposes, where it generally (and arguably) is of greatest concern to identify those who fare least well. Increasing the number of items and/or modifying the response scale from a dichotomous to a polytomous one [47] may provide means of improving and expanding the scale's usefulness.

The sample used here was drawn from earlier studies not designed for the present purpose. However, we do not consider this a major problem since the Rasch model enables scale items to be examined in a way that is freed from the characteristics of the study sample. Another limitation could be the concurrent use of multiple questionnaires in some of the original studies and the fact that people did not respond to the 24-item NHPD but to the 38-item NHP, from which NHPD data were derived. This may, hypothetically, have influenced responses and, hence, psychometric performance. However, this strategy is a common procedure in psychometric studies and has generally not been found problematic [48-50]. Nevertheless, further studies using only the NHPD and not the full NHP are warranted. Furthermore, our data did not allow us to address some important measurement properties such as test-retest stability and responsiveness. Finally, this study only considered two diagnostic groups. Additional analyses in other patient populations are needed to further determine the generic properties of the NHPD.

## Conclusion

The NHPD displayed good measurement properties among people with PD and PAD but exhibited varying degrees of DIF by diagnosis for seven items. Although this DIF may represent some degree of multidimensionality, it did not have a clinically significant impact on the total score. This supports the generic measurement properties of the NHPD as a sufficiently unidimensional survey tool among people with PD and PAD. These results should encourage others to consider using the NHPD as a simple patient-reported index of illness-related distress in chronic disabling disorders and to reassess available NHP data within the NHPD framework to further evaluate its strengths and weaknesses.

## Competing interests

The authors declare that they have no competing interests.

## Authors' contributions

CWH conducted literature searches, participated in designing the study, data analyses and interpretation, and drafting of the manuscript. RK participated in designing the study and drafting of the manuscript. PH conceptualized and participated in designing the study, conducted the analyses and drafted the manuscript. All authors collected data and read and approved the final manuscript.

## Supplementary Material

Additional file 1 Overall fit statistics for the NHPD following successive split of NHPD items displaying signs of DIF between PD and PAD. Step-by-step changes in mean item fit residual values and total item-trait chi-square statistics during successive split of of NHPD items displaying signs of DIF between people with PD and PAD. Click here for file
