# Supplementary material for: Cross-diagnostic validity of the Nottingham health profile index of distress (NHPD)
Source: Health Qual Life Outcomes. 2008 Jul 2;6:47. doi: 10.1186/1477-7525-6-47 (PMC2483964; doi:10.1186/1477-7525-6-47)
Supplement: Additional file 1 — Overall fit statistics for the NHPD following successive split of NHPD items displaying signs of DIF between PD and PAD. Step-by-step changes in mean item fit residual values and total item-trait chi-square statistics during successive split of of NHPD items displaying signs of DIF between people with PD and PAD. [file 1477-7525-6-47-S1.doc]

## Additional file 1 - Overall fit statistics for the NHPD following successive split of NHPD items displaying signs of DIF between PD and PAD ^a^

|  | Item fit residual | | Total item-trait interaction | |  |
| --- | --- | --- | --- | --- | --- |
| Items split by diagnosis | Mean ^b^ | SD ^c^ | Chi-square (df) | P-value | Reliability ^d^ |
| - | -0.571 | 1.416 | 134.337 (120) | 0.175 | 0.841 |
| 6 | -0.581 | 1.416 | 163.899 (125) | 0.011 | 0.843 |
| 6, 11 | -0.560 | 1.308 | 157.382 (130) | 0.051 | 0.845 |
| 6, 11, 17 | -0.535 | 1.312 | 167.201 (135) | 0.032 | 0.844 |
| 6, 11, 17, 7 | -0.514 | 1.312 | 166.727 (140) | 0.061 | 0.843 |
| 6, 11, 17, 7, 4 | -0.496 | 1.296 | 163.765 (145) | 0.136 | 0.843 |
| 6, 11, 17, 7, 4, 18 | -0.506 | 1.275 | 182.506 (150) | 0.036 | 0.843 |
| 6, 11, 17, 7, 4, 18, 8 | -0.488 | 1.250 | 188.538 (155) | 0.034 | 0.844 |

^a^ Conducted in order of degree of DIF, as defined by the F-statistics.

^b^ Should be close to 0.

^c^ Should be close to 1.

^d^ Index of person separation, a Rasch based reliability statistic analogous to Cronbach’s alpha/KR-20.

NHPD, Nottingham Health Profile index of Distress; DIF, differential item functioning; PD, Parkinson’s disease; PAD, peripheral arterial disease; SD, standard deviation; df, degrees of freedom.
